# Image based deep learning in 12-lead ECG diagnosis

**DOI:** 10.3389/frai.2022.1087370

**Published:** 2023-01-09

**Authors:** Raymond Ao, George He

**Affiliations:** ^1^The Prince Charles Hospital, Chermside, QLD, Australia; ^2^Royal Prince Alfred Hospital, Sydney, NSW, Australia; ^3^Faculty of Medicine and Health, The University of Sydney, Sydney, NSW, Australia

**Keywords:** ECG, diagnosis, classification, deep learning, 12-lead ECG

## Abstract

**Background:**

The electrocardiogram is an integral tool in the diagnosis of cardiovascular disease. Most studies on machine learning classification of electrocardiogram (ECG) diagnoses focus on processing raw signal data rather than ECG images. This presents a challenge for models in many areas of clinical practice where ECGs are printed on paper or only digital images are accessible, especially in remote and regional settings. This study aims to evaluate the accuracy of image based deep learning algorithms on 12-lead ECG diagnosis.

**Methods:**

Deep learning models using VGG architecture were trained on various 12-lead ECG datasets and evaluated for accuracy by testing on holdout test data as well as data from datasets not seen in training. Grad-CAM was utilized to depict heatmaps of diagnosis.

**Results:**

The results demonstrated excellent AUROC, AUPRC, sensitivity and specificity on holdout test data from datasets used in training comparable to the best signal and image-based models. Detection of hidden characteristics such as gender were achieved at a high rate while Grad-CAM successfully highlight pertinent features on ECGs traditionally used by human interpreters.

**Discussion:**

This study demonstrates feasibility of image based deep learning algorithms in ECG diagnosis and identifies directions for future research in order to develop clinically applicable image based deep-learning models in ECG diagnosis.

## 1. Introduction

The electrocardiogram (ECG) is an essential tool in diagnoses of cardiovascular diseases which are a leading cause of death worldwide (Collaborators GBDCoD, 2018). As ECGs have transitioned from analog to digital, automated computer analysis has gained traction and success in diagnoses of medical conditions (Willems et al., 1987; Schlapfer and Wellens, 2017). Deep learning methods have shown excellent diagnostic performance on classifying ECG diagnoses using signal data, even surpassing individual cardiologist performance in some studies. For example, one study which used raw ECG data created a deep neural network (DNN) which performed similarly to or better than the average of individual cardiologists in classifying 12 different rhythms, including atrial fibrillation/flutter, atrioventricular block, junctional rhythm, and supra/ventricular tachycardia in single lead ECGs (Hannun et al., 2019). Other studies used signal data from 12-lead ECGs with excellent results in arrhythmia classification (Baek et al., 2021).

While automated diagnoses of ECGs provide great promise in improving workflow many, developed models have focused on the diagnosis of singular clinical pathology, limiting utility as ECGs may have multiple abnormalities simultaneously (Biton et al., 2021; Raghunath et al., 2021). Further, most tools are based off analysis of raw signal data (Hannun et al., 2019; Hughes et al., 2021; Sangha et al., 2022). This presents a challenge for models in many areas of clinical practice where ECGs are printed on paper or only digital images are accessible, especially in remote and regional settings where often there is the largest lack of access to speciality medical opinion (Schopfer, 2021). In such areas, imaged based deep learning models for ECG recognition would serve best of which there are few studies in the literature. One study developed an image based model to differential between normal or abnormal ECGs while another achieved 99.05% average accuracy and 97.85% average sensitivity for 7 cardiac conditions based off analysis of individual ECG beats (Jun et al., 2018). A recent paper created a model superior to signal based imaging achieving area under the received curve (AUROC) of 0.99 and area under Precision-Recall curve (AUPRC) 0.86 for 6 clinical disorders (Sangha et al., 2022).

To the best of our knowledge, this study is the first to train a convolutional neural network (CNN) capable of classifying raw images of 12-lead ECGs for 10 pathologies. The method used in this experiment differs from most other studies in that ECG image data is directly used to train and test deep learning models as opposed raw signal data or transformations of signal data.

## 2. Materials and methods

### 2.1. Datasets

The primary dataset used for model development and evaluation was PTB-XL. We also tested for external validity of models across different unseen datasets, as well as with different datasets in combination.

The following publicly available ECG datasets were used:

- PTB-XL (PTB) (Wagner et al., 2020).
- CPSC 2018 database (CPSC) (Liu et al., 2018).
- 12-lead ECG database for arrhythmia research from Chapman University and Shaoxing People's Hospital (Shaoxing) (Zheng et al., 2020).
- Test dataset for: Automatic multi-label ECG diagnosis of impulse or conduction abnormalities in patients with deep learning algorithm: a cohort study (Tongji) (Zhu et al., 2020).

Each dataset contained raw 10-s 12-lead ECG signal waveform data with corresponding diagnostic labels.

### 2.2. Data pre-processing

**Figure 1:**
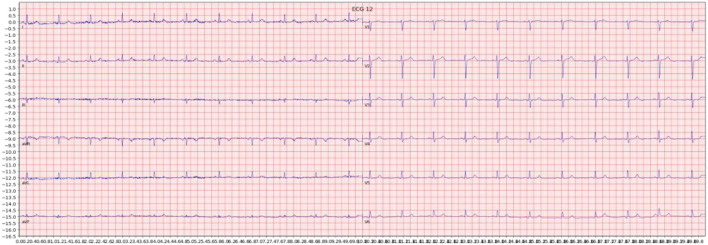
Example of an ECG image generated from the signal data of one sample in the dataset.

For each individual ECG sample, an image was generated by plotting the signal data. We used the Python ECG plot library (dy1901, 2022), which generates 12-lead ECG images resembling ECG displays and print-outs commonly used in clinical practice. An example is shown in Figure 1. Images were processed at 1,600 × 512 resolution.

**Figure 2:**
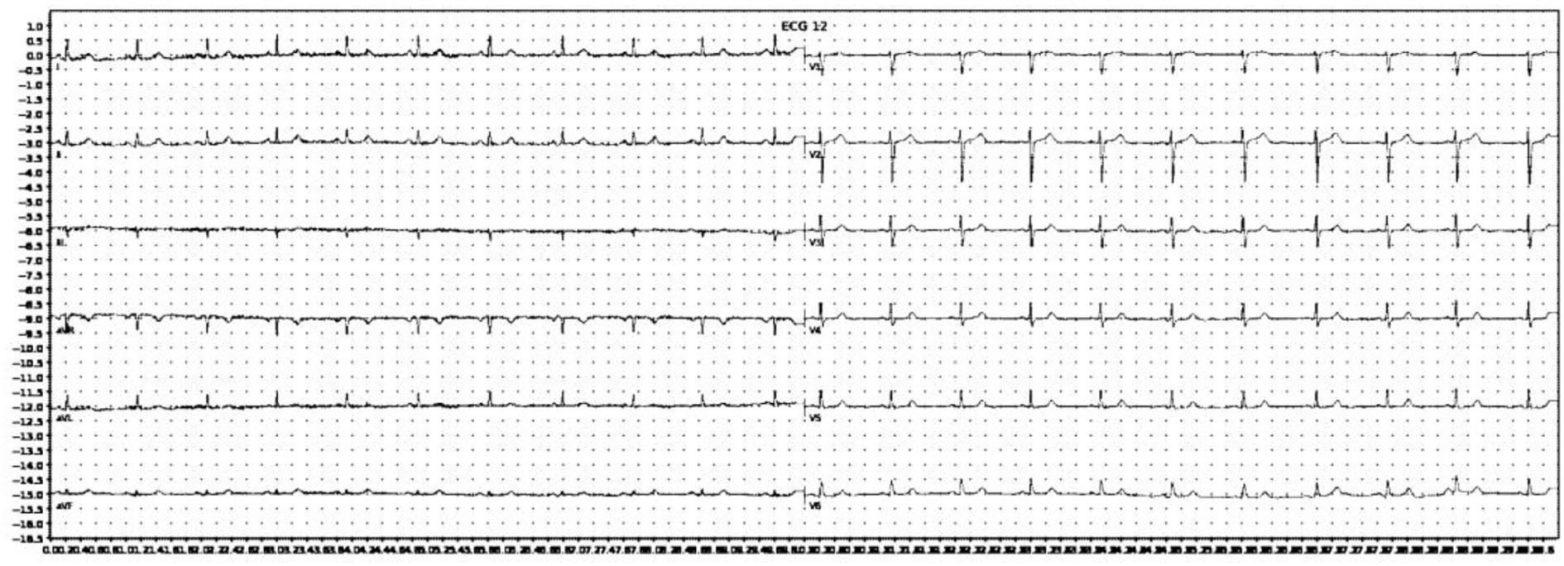
Example of an ECG image after grayscale conversion and adaptive thresholding.

The images were then converted to grayscale, then binarised using simple thresholding, Otsu thresholding, and adaptive thresholding. A binarised copy was saved for each of the thresholding techniques. For further data augmentation, a slightly blurred vision of the grayscale image was created, and the aforementioned thresholding techniques were also applied. In total, eight augmented copies of each original ECG image was generated. An example of an image after grayscale conversion and adaptive thresholding is shown in Figure 2.

### 2.3. Classification task overview

Binary classification models were trained predict the presence and absence of:

- Normal ECG (NORM).
- Left bundle branch block (LBBB).
- Right bundle branch block (RBBB).
- Atrial fibrillation (AFIB).
- Atrial flutter (AFLT).
- First degree AV block (fAVB).
- Myocardial infarction (MI).
- Wolff-Parkinson White (WPW).
- Supraventricular tachycardia.

Models were trained using the PTB-XL dataset and evaluated on holdout test data from PTB-XL (Table 1). Additionally, models were also tested on ECG images from other datasets not involved in training. Further testing was done on combined datasets, where matching diagnostic labels were present (Table 2).

**Table 1:** Test results of models trained on PTB-XL ECGs and tested on a holdout test set from PTB-XL.

**Diagnosis**	**AUPRC**	**AUROC**	**Sensitivity**	**Specificity**
AFIB	0.999	1.000	0.992	0.999
AFLT	0.985	0.989	1.000	0.964
AMI	0.9895	0.991	0.977	0.944
AVB	0.979	0.985	0.969	0.940
IMI	0.981	0.983	0.948	0.9162
LMI	0.540	0.511	0.349	0.632
LVH	0.934	0.971	0.897	0.954
MI	0.988	0.989	0.960	0.940
NORM	0.997	0.998	0.980	0.980
PVC	0.984	0.986	0.950	0.970
RBBB	1.000	1.000	0.996	1.000
sAVB	0.860	0.960	0.800	0.933
Sex (Female)	0.997	0.996	0.985	0.954
SVT	1.000	1.000	0.875	1.000
tAVB	0.848	0.946	1.000	0.867

AFIB, atrial fibrillation; AFLT, atrial flutter; AMI, anterior myocardial infarction; AVB, atrioventricular block; IMI, inferior myocardial infarction; LMI, lateral myocardial infarction; LVH, left ventricular hypertrophy; MI, myocardial infarction; NORM, normal; PVC, premature ventricular contractions; RBBB, right bundle branch block; sAVB, 2^*nd*^ degree atrioventricular block; SVT, supraventricular tachycardia; tAVB, total atrioventricular block.

**Table 2:** Test results of models trained on combined datasets and tested on holdout data from the combined datasets.

**Diagnosis**	**Dataset combination**	**AUPRC**	**AUROC**	**Sensitivity**	**Specificity**
AFIB	PTB + CPSC + Shaoxing	0.994	0.999	0.970	1.00
fAVB	PTB + CPSC + Shaoxing	0.985	0.987	0.95	0.960
LBBB	PTB + CPSC + Shaoxing	0.987	0.999	0.940	1.000
RBBB	PTB + CPSC + Shaoxing	0.298	0.739	0.460	0.850
MI	PTB + Shaoxing	0.954	0.987	0.800	0.990
SVT	PTB + Shaoxing	0.980	1.000	0.950	1.000
WPW	PTB + Shaoxing + Tongji	0.756	0.842	0.930	0.370

AFIB, atrial fibrillation; fAVB, 1st degree AV block; LBBB, left bundle branch block; RBBB, right bundle branch block; MI, myocardial infarction; SVT, supraventricular tachycardia; WPW, Wolf Parkinson White.

For each training run, the included samples from all datasets were randomly shuffled, and split into training, validation and holdout test sets, with splits of 0.8, 0.1, and 0.1 respectively.

### 2.4. Model architecture and training

Model were built on VGG16 architecture with Imagenet pre-trained weights. The original classification layer was removed and replaced with a classification head consisting of a global average pooling 2D layer, a dropout layer for training, followed by a fully connected layer with one output and sigmoid activation.

The classification head was initially trained for up to 10 epochs with early stopping, while all other layers were frozen. The entire model was then unfrozen, and trained until no further drop in validation loss was seen (early stopping with patience of 6). The learning rate was 1 × 10^∧^-5. A learning rate schedule involving reducing the learning rate when the validation loss plateaued was trialed, without significant improvement of results. For most training instances, binary cross-entropy loss was used. We also experimented with focal loss for highly imbalanced datasets.

## 3. Results

The models demonstrated good performance when tested on unseen holdout test data from the original datasets used on training. Generalization to unseen, external datasets was poorer. Performance of models trained on a combination of different datasets mixed together showed good performance on holdout test splits containing the mixed datasets. The results are summarized in table below.

### 3.1. Visual explanation

**Figure 3:**
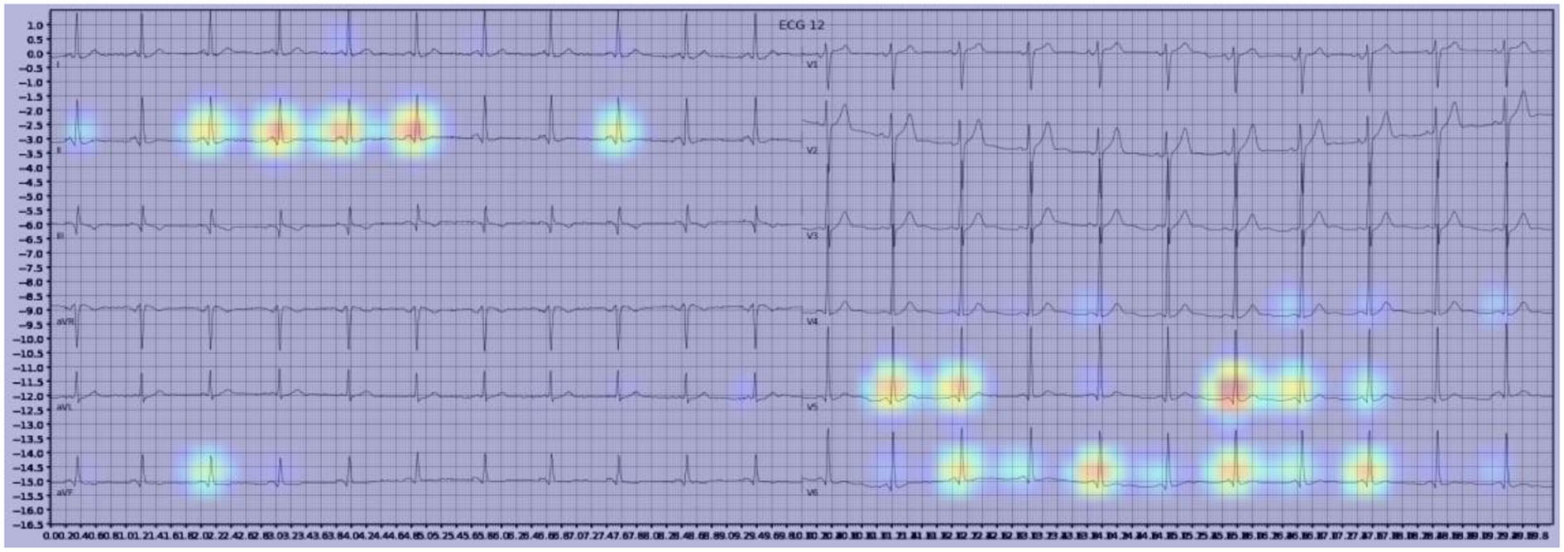
Example of an activation heatmap for an ECG showing WPW.

**Figure 4:**
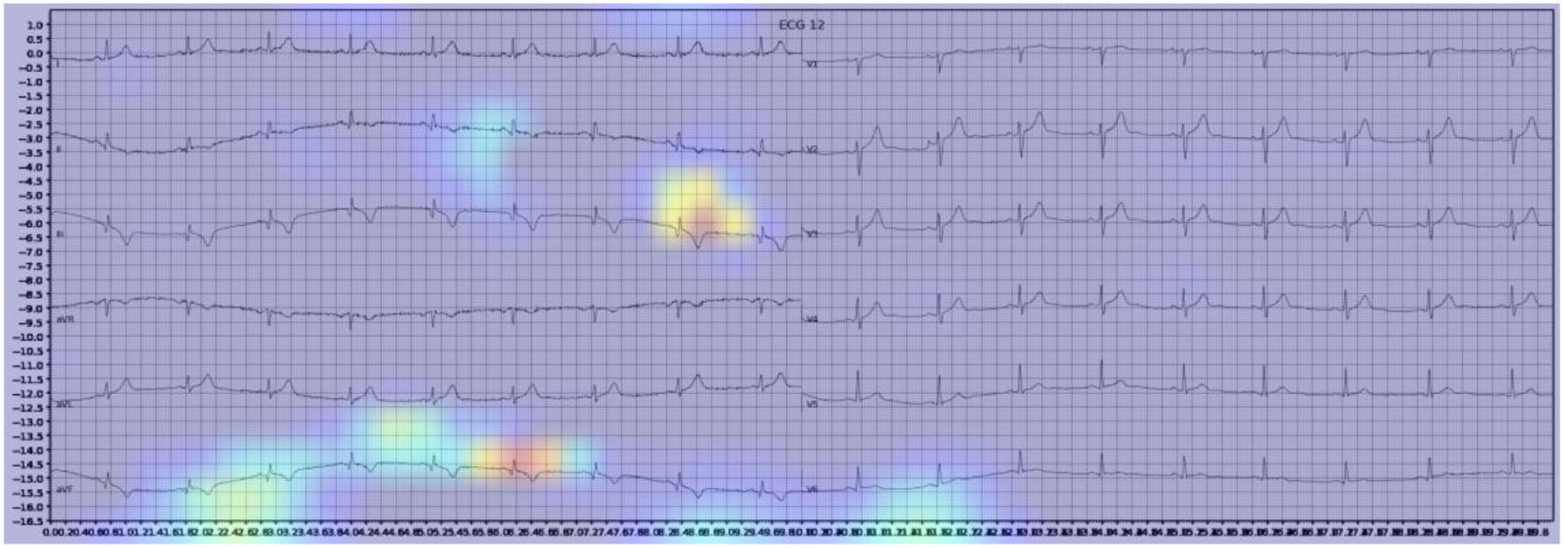
Example of an activation heatmap for an ECG showing myocardial infarction.

**Figure 5:**
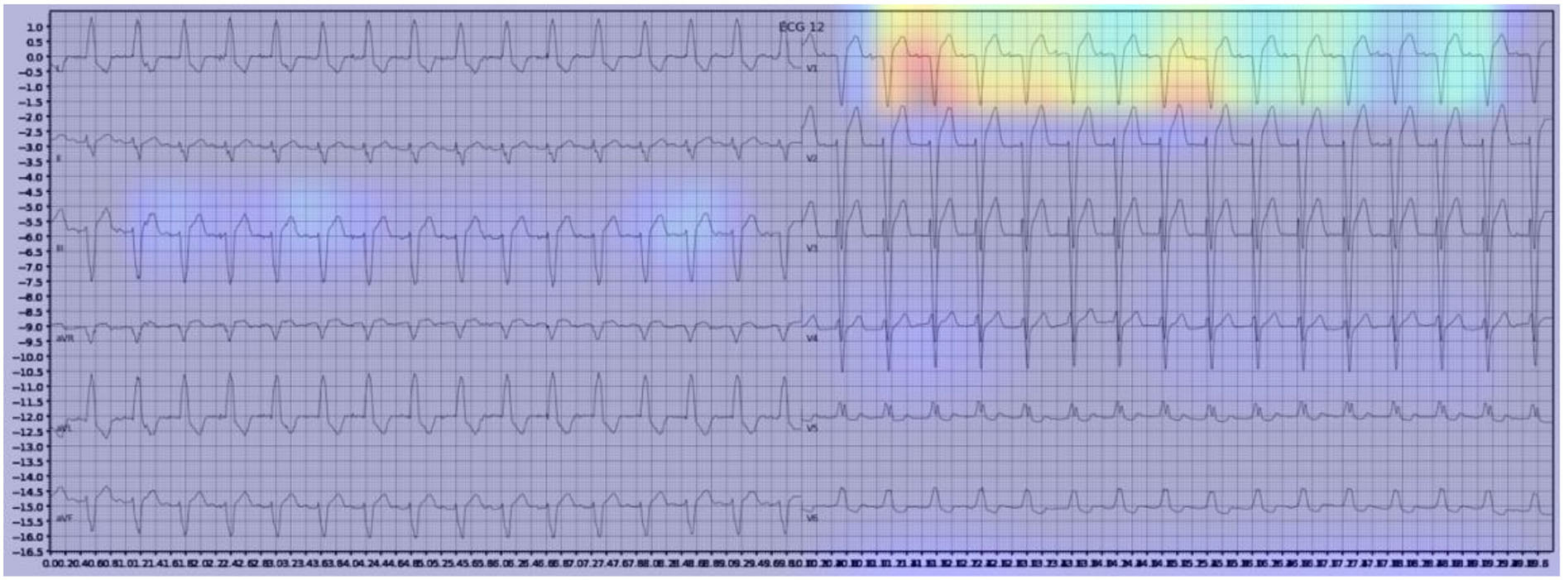
Example of an activation heatmap for an ECG showing LBBB.

Gradient-weighted Class Activation Mapping (Grad-CAM) creates a heatmap to visualize areas of the image which are important in predicting its class. A few examples are illustrated below with Figure 3 demonstrating delta waves in WPW, Figure 4 demonstrating ST segment changes in MI and Figure 5 highlighting deep broad S waves in V1 for LBBB.

## 4. Discussion

Our model demonstrated strong diagnostic performance on unseen ECGs sampled from the same population(s) and dataset(s) as that used for model training. This high level of internal validity has been reflected in the literature from previous computer vision-based models (Mohamed et al., 2015; Jun et al., 2018; Sangha et al., 2022). Jun et al. using a two-dimensional CNN detected different individual ECG beats to examine 7 unique cardiac arrhythmias while Sangha et al. created a model based on Efficientnet B3 architecture to examine 6 disorders with equivalent to superior performance compared to signal-based methods. In comparison, our model examined up to 13 diagnoses, was built on combinations of datasets and evaluated more datasets when examining external validity. Table 3 shows results from this model were comparable to the other recent image-based studies and 12-lead ECG signal based work. Additionally, some of the most accurate single pathology signal-based models e.g., for atrial fibrillation have comparable findings to this study (AUROC 0.997 vs. 1 and Sensitivity of 0.985 to 0.992) (Jo et al., 2021). While demonstrating comparable accuracy, this study improves upon several signal-based studies in the literature focusing on individual pathologies or single leads that are consequently limited with 12 lead readings as well as ECGs with multiple pathologies (Javadi et al., 2013; Biton et al., 2021; Raghunath et al., 2021).

**Table 3:** Comparison of image and signal based ECG recognition algorithms.

**Work**	**Architecture**	**Database**	**Diagnoses**	**Label**	**AUROC**	**AUPRC**	**Sens**	**Spec**
Mohamed et al. (2015)	Multilayer Perceptron (MLP)	Random sample *N* = 20	Normal, abnormal	Cardiac pathology	Not reported	Not reported	0.99	0.99
Jun et al. (2018)	VGGNet 2D CNN	MIT-BIH *N* = 106,501	Normal, LBBB, RBBB, PVC, PB, APC, VF, VEB	Cardiac pathology	0.989	Not reported	0.978	0.996
Sangha et al. (2022)	EfficientNet B3	TNMG *N* = 2,228,236	Normal, LBBB, RBBB, AF, fAVB, SB, ST		Hold out set
				Cardiac pathology	0.992	0.725	0.817	0.992
				Gender	0.934	0.905	0.849	0.875
					Cardiologist validated set
				Cardiac pathology	0.997	0.915	0.930	0.995
				Gender	0.890	0.845	0.798	0.826
					External set (PTB–XL)
				Cardiac pathology	0.981	0.776	0.805	0.989
				Gender	0.899	0.904	0.876	0.738
					Real world–lake regional hospital
				Cardiac pathology	0.984	0.935	0.909	0.980
					Real world–web based
				Cardiac pathology	0.932	0.799	0.905	0.949
				Gender	0.778	0.784	0.909	0.516
This study	VGG16	PTB-XL *N* = 21,801	Normal, LBBB, RBBB, AF, AFLT, fAVB, AMI, IMI, LMI, WPW, SVT, sAVB, tAVB, PVC, LVH		Hold out set
				Cardiac pathology	0.982	0.967	0.950	0.954
				Gender	0.996	0.997	0.985	0.954
**Signal based algorithms**−**12-lead ECG**								
Ribeiro et al. (2020)		TNMG *N* = 2,228,236	fAVB, RBBB, LBBB, SB, AF, ST	Cardiac pathology	Not reported	Not reported	0.935	0.997
Zhu et al. (2020)		3 Clinical sites *N* = 180,112	AF, AFLT, JR, PVC, IVR, VT, PB, LBBB, WPW	Cardiac pathology	0.983	Not reported	0.867	0.995

fAVB, 1st degree AV block; sAVB, 2^*nd*^ degree AV block; tAVB, total AV block; AF, atrial fibrillation; AFLT, atrial flutter; AMI, anterior myocardial infarction; IMI, inferior myocardial infarction; LMI, lateral myocardial infarction; APC, atrial premature contraction beat; AUPRC, area under precision recall curve; AUROC, area under receiver operator characteristic curve; IVR, idioventricular rhythm; JR, junctional rhythm; LBBB, left bundle branch block; NPV, negative predictive value; PB, paced beat; PPV, positive predictive value; PVC, premature ventricular contractions; RBBB, right bundle branch block; SB, sinus bradycardia; ST, sinus tachycardia; SVT, supraventricular tachycardia; VEB, ventricular escape beat; VF, ventricular flutter wave beat; WPW, Wolf Parkinson White; LVH, left ventricular hypertrophy; AUROC, area under the received curve; AUPRC, area under Precision-Recall curve; Sens, sensitivity; Spec, specificity.

A great advantage presented by our model is that current deep learning tools primarily rely in signal data which has not been optimized for lower resources setting such as a rural and remote environment. A large majority of ECGs in current practice are either printed or scanned as images which limits the utility of signal-based models. Additionally, while many models have been designed to accurately detect individual disorders, ECGs with multiple co-existing abnormalities present a challenge. Further, the model also demonstrates consistent and superior performance compared to previous image-based studies in identifying the gender of patients from ECGs from both internal and external datasets, suggesting that hidden features can be recognized accurately as demonstrated in signal-based models (Attia et al., 2019; Kim and Pyun, 2020). This provides great promise as ECG data is increasingly collected with other observations and vital signs which may be utilized *via* algorithms.

**Figure 6:**
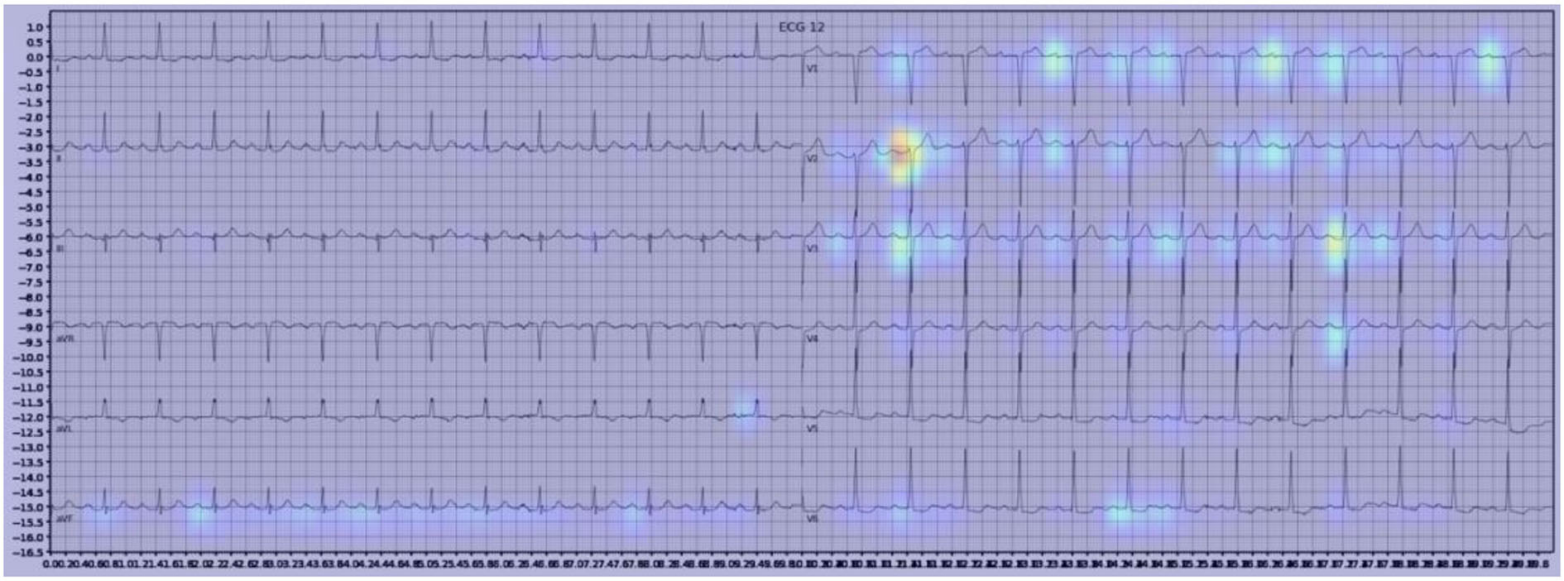
Example of an activation heatmap for an ECG showing First Degree AV Block.

Our model incorporated the use of Gradient-weighted Class Activation Mapping (Grad-CAM) to highlight the regions in an image predicting a given label. This also allowed evaluation of whether the assigned labels identified clinically relevant information or were founded in heuristics from spurious data features (DeGrave et al., 2020). We found there was large correspondence with features used in human interpretation of ECGs. For example, Grad-CAM highlighted delta waves in WPW (Figure 3), ST segment changes in MI (Figure 4), deep broad S waves in V1 for LBBB (Figure 5), prolonged PR segments in 1st deg AV block (Figure 6), and QRS without P waves in AF, and. In few cases, it was less relatable to human diagnosis, e.g., highlighting the area following an ectopic beat rather than the abnormally large QRS complexes which would normally stand out to human interpreters. These occurred in a small percentage and may be improved on using more model training across a variety of data sets or integrating other technologies such as HiResCAM (Draelos and Carin, 2020). In application, by presenting a heatmap, it provides context and evidence demonstrating how the diagnosis was achieved. This allows the clinician to make a visually informed decision about the algorithm diagnosis assisting in potential better integration into routine clinical practice (Makimoto et al., 2020).

In terms of computational complexity, our study had PC specifications of Ryzen 9 5900x CPU, RTX 3080 and 3080 Ti, and 64 GB RAM running on Linux Mint. Training times took from 18 to 36 h for fine tuning of VGG 16 for binary classification of each diagnosis label individually, until stopped by the early stopping callback based on plateauing validation AUROC. Inference times were in the range of 3–5 s per diagnosis label for each image. Although not previously reported in imaged based algorithm studies, it demonstrates that while model training can be relatively time consuming to train, the output is reached in a timely manner. Previously, computerized interpretations of ECGs have been shown to reduce analysis times and such findings highlights the potential for incorporation of computer vision algorithms into routine clinical care as an adjunct for diagnosis and decision making (Schlapfer and Wellens, 2017).

### 4.1. Future work and limitations

The models were not as accurate when applied unseen external datasets-this may be due to differences in labeling criteria for diagnoses between the datasets, or variances in ECG quality. Nonetheless, some diagnostic labels were accurately classified with unseen external datasets (e.g., LBBB, RBBB and WPW) as shown in Table 4, which shows that this technology has the potential to be useful on unseen datasets, given a sufficient amount of training data and more consistent labeling of training data. Accuracy could be better improved with further pre-processing steps such as shuffling positions of different leads on the image to help the model learn the relevance of different leads, and inclusion of even more different independent datasets. We could also explore the effect of adding additional clinical information known to clinicians at time of ECG interpretation, such as age, gender, weight and height and evaluate accuracy with such data and image-based algorithms could bolster disease stratification models. Combination in architectures between papers may further hold superior results.

**Table 4:** Test results of models tested on separate, unseen datasets than those used in training.

**Diagnosis**	**Training datasets**	**External datasets**	**AUPRC**	**AUROC**	**Sensitivity**	**Specificity**
AFIB	PTB + CPSC	Tongji	0.203	0.766	0.510	0.880
AFIB	PTB + CPSC	Shaoxing	0.231	0.613	0.760	0.400
AFIB	PTB + CPSC	Shaoxing	0.582	0.842	0.640	0.940
AFIB	PTB + CPSC + Shaoxing	Tongji	0.080	0.549	0	1.000
fAVB	PTB + CPSC + Shaoxing	Tongji	0.249	0.824	0.5	0.920
fAVB	PTB + CPSC + Shaoxing	Georgia	0.697	0.956	0.790	0.940
LBBB	PTB	Testcohort	1.000	1.000	0.996	1.000
MI	PTB	Georgia	0.308	0.868	1.000	0.270
MI	PTB	Shaoxing + Georgia	0.374	0.888	0.950	0.310
MI	PTB	Shaoxing	0.403	0.908	0.940	0.350
NORM	PTB	Testcohort	0.187	0.867	0.690	0.850
NORM	PTB	Shaoxing	0.247	0.728	0.420	0.810
NORM	PTB	CPSC	0.415	0.874	0.770	0.820
PVC	PTB	Tongji	0.461	0.889	0.820	0.840
RBBB	PTB	CPSC	0.934	0.971	0.8967	0.954
WPW	PTB + Shaoxing	Tongji	0.751	0.945	0.820	0.920

AFIB, atrial fibrillation; fAVB, 1st degree AV block; LBBB, left bundle branch block; MI, myocardial infarction; NORM, normal; PVC, premature ventricular contractions; RBBB, right bundle branch block; WPW, Wolf Parkinson White.

## 5. Conclusion

This research demonstrates that computer vision AI models can diagnose conditions on ECG with good accuracy. Future research which could bring this technology closer to clinical application could focus on developing models which can generalize to a wide range of ECG image formats from various sources and cover a wider range of relevant clinical diagnoses. Additional diagnoses which would be of interest clinically would include diagnosing STEMI in patients with LBBB or pacemaker, differentiating SVT with aberrancy vs. VT, and specific subtypes of AV block. Furthermore, models could be developed to be applicable to different ECG formats or styles. The techniques demonstrated here could also be applied for novel practical applications, such as smartphone applications to diagnose photos of ECGs, or in telehealth. Overall, classification performance on ECG images using deep CNNs is comparable to the best models using raw ECG signal holdout test data from the same dataset.

## Data availability statement

The original contributions presented in the study are included in the article/supplementary material, further inquiries can be directed to the corresponding author.

## Ethics statement

Ethical review and approval was not required for the study on human participants in accordance with the local legislation and institutional requirements. Written informed consent for participation was not required for this study in accordance with the national legislation and the institutional requirements.

## Author contributions

RA was involved in data processing, training, and evaluating machine learning models. GH was involved in literature review and editing. All authors contributed to the article and approved the submitted version.
